# Antimicrobial Activity of Tannic Acid *In Vitro* and Its Protective Effect on Mice against Clostridioides difficile

**DOI:** 10.1128/spectrum.02618-22

**Published:** 2022-12-20

**Authors:** Weigang Wang, Jing Cao, Jing Yang, Xiaoran Niu, Xiaoxuan Liu, Yu Zhai, Cuixin Qiang, Yanan Niu, Zhirong Li, Ning Dong, Baojiang Wen, Zirou Ouyang, Yulian Zhang, Jiayiren Li, Min Zhao, Jianhong Zhao

**Affiliations:** a The Second Hospital of Hebei Medical University, Shijiazhuang, Hebei, China; b Hebei Provincial Center for Clinical Laboratories, Shijiazhuang, Hebei, China; Tainan Hospital, Department of Health, Executive Yuan

**Keywords:** *Clostridioides difficile*, antibiotics, tannic acid, spore, toxin, mouse model, microbiota

## Abstract

Clostridioides difficile infection (CDI), recurrently reported as an urgent threat owing to its increased prevalence and mortality, has attracted significant attention. As the use of antibiotics to treat CDI has many limitations, such as high recurrence rate, the need to actively seek and develop other drugs that can effectively treat CDI with fewer side effects has become a key issue in CDI prevention and treatment. This study aimed to evaluate the inhibitory effect of Galla chinensis (GC) and its main component, tannic acid (TA), against C. difficile *in vitro* and its therapeutic effect on CDI *in vivo*. When GC and TA concentrations were 250 and 64 mg/L, respectively, the cumulative antibacterial rate against C. difficile reached 100%. The sub-MIC of TA significantly inhibited C. difficile sporulation, toxin production, and biofilm formation *in vitro*. Compared with the CDI control group, TA-treated mice lost less weight and presented a significantly improved survival rate. TA significantly reduced the number of spores in feces, decreased serum TcdA level, and increased serum interleukin 10 (IL-10). Based on the inhibitory effect of TA on C. difficile *in vitro* and its therapeutic effect on the CDI mouse model, we consider TA as a potentially effective drug for treating CDI.

**IMPORTANCE** Clostridioides difficile is one of the major pathogens to cause antibiotic-associated diarrhea. Although antibiotic treatment is still the most commonly used and effective treatment for CDI, the destruction of indigenous intestinal microbiota by antibiotics is the main reason for the high CDI recurrence rate of about 20%, which is increasing every year. Moreover, the growing problem of drug resistance has also become a major hidden danger in antibiotic treatment. GC has been used to treat diarrhea in traditional Chinese medicine. In the present study, we evaluated the inhibitory effect of TA, the main component of GC, on dissemination and pathogenic physiological functions of C. difficile *in vitro*, as well as its therapeutic efficacy in a CDI model. Overall, TA is considered to be a potentially effective drug for CDI treatment.

## INTRODUCTION

Clostridioides difficile is an obligate anaerobic and spore-producing Gram-positive bacterium and a leading cause of antibiotic-associated diarrheal disease (1). With the emergence of highly toxic and antibiotic-resistant strains, C. difficile infection (CDI) has attracted significant attention (2). According to the latest treatment guidelines of the Infectious Diseases Society of America (IDSA), oral vancomycin (VAN) or fidaxomicin is indicated as the first-line treatment option, with oral metronidazole listed as an alternative (3). Although antibiotic therapy is a regular treatment method, its long-term use can lead to intestinal microbiota disorders and drug resistance, which become the driving force behind the high recurrence rate of CDI (4). Therefore, along with rational use of antibiotics for the prevention and treatment of CDI, there is an urgent need to actively seek and develop drugs that can effectively treat CDI with fewer side effects (5).

Galla chinensis (GC) has been used to treat diarrhea, dysentery, hemorrhoids, periodontitis, dental caries, and other diseases, and its anti-inflammatory and antibacterial effects are the basis of its effective treatment (6–10). The main active ingredients of GC are tannic acid (TA), gallic acid (GA), gall oil, etc. (11, 12). TA has many biological functions such as antibacterial, anti-inflammatory, intestinal astringency, anticancer, antioxidation, and lipid-lowering properties (13–17). This study is the first to evaluate the *in vitro* antimicrobial activity of GC and its main component, TA, on C. difficile. Also, the inhibitory effect of TA on the spore formation, toxin production, and biofilm formation of C. difficile was evaluated *in vitro*. Furthermore, the therapeutic effect of TA was estimated in CDI model mice.

## RESULTS

### MICs of GC and TA against C. difficile.

**FIG 1:**
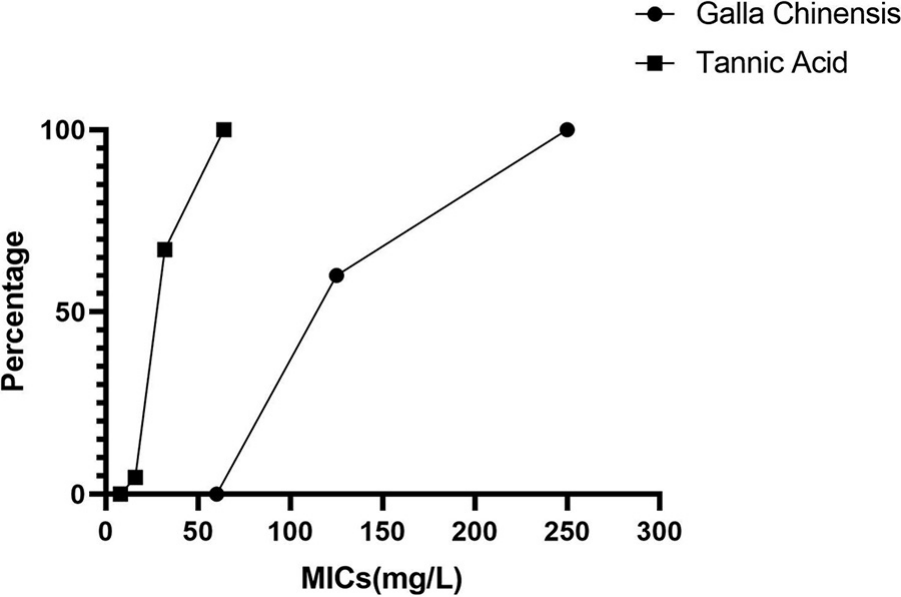
Cumulative antibacterial percentage of Galla chinensis and tannic acid against C. difficile.

All the clinical isolates were identified as C. difficile by matrix-assisted laser desorption ionization–time of flight mass spectrometry (MALDI-TOF MS) and 16S rRNA gene sequencing. The MICs of GC against 130 C. difficile strains were clustered in concentrations of 125 and 250 mg/L, and the cumulative antibacterial percentage reached 100% at 250 mg/L. The MIC values of 125 and 250 mg/L GC were effective against 78 (60%) and 52 (40%) C. difficile strains, respectively (Fig. 1).

The MICs of TA against the 152 C. difficile strains were clustered in three concentrations of 16, 32, and 64 mg/L, and the cumulative antibacterial percentage reached 100% at 64 mg/L TA (Fig. 1). The MIC values of 16, 32, and 64 mg/L TA were effective against 7 (4.6%), 95 (64.5%), and 50 (32.9%) C. difficile strains, respectively, while those of GA against 152 C. difficile strains examined in this study were all >256 mg/L, indicating that GA did not exert an effective antibacterial effect in the concentration range examined in the present study. Therefore, we presumed that TA in GC is efficient in inhibiting C. difficile.

The MICs of TA and vancomycin against ATCC BAA-1870 and BAA-1382 were determined by the agar dilution method. The results revealed that the TA MIC against the two C. difficile test strains was 64 mg/L, whereas the vancomycin MICs against ATCC BAA-1870 and BAA-1382 were 1 and 0.5 mg/L, respectively.

### TA inhibits sporulation of C. difficile.

**FIG 2:**
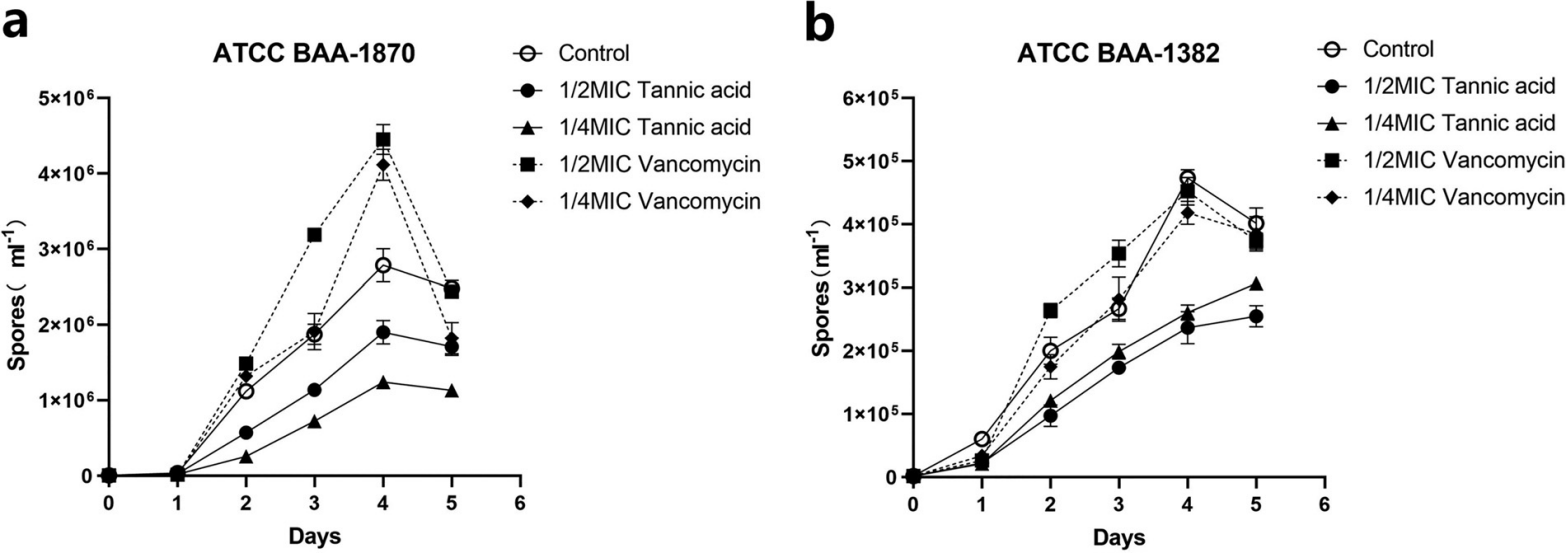
Effect of 1/2× MIC and 1/4× MIC tannic acid (TA) and vancomycin (VAN) on sporulation of C. difficile ATCC BAA-1870 (a) and BAA-1382 (b). Each experiment was repeated twice independently and included six biological replicates. Error bars represent standard error of the mean (SEM). The *P* value was obtained by one-way ANOVA.

At the five time points of the experiment, compared with the untreated control group, both 1/2× MIC and 1/4× MIC TA treatments significantly inhibited spore formation of C. difficile (*P < *0.05). However, the control drug vancomycin failed to inhibit the spore-forming ability of the test strains. Furthermore, the inhibitory effect of TA on the spore-forming ability of C. difficile also differed among the strains (Fig. 2).

### TA reduces C. difficile toxin production.

**FIG 3:**
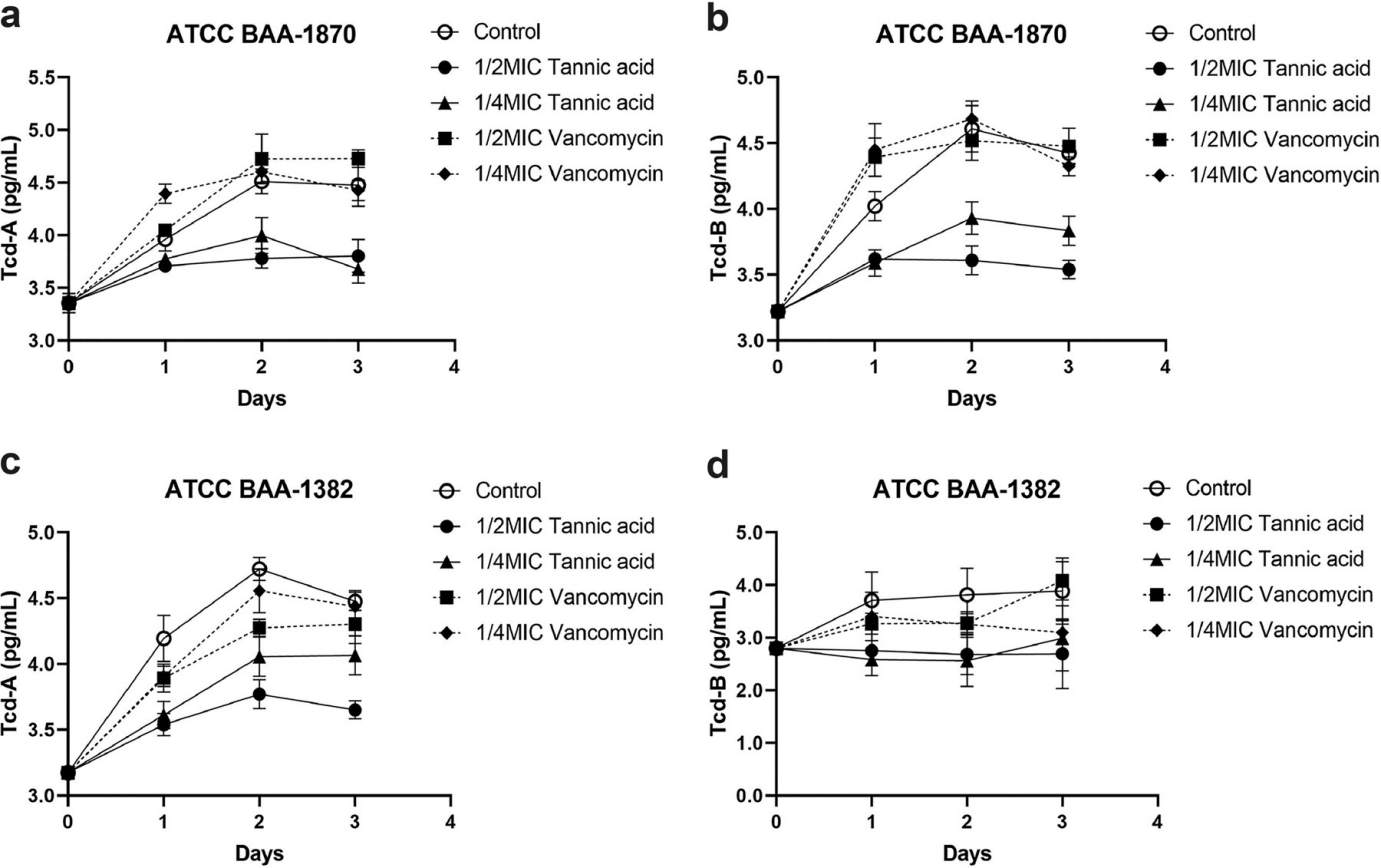
Effect of 1/2× MIC and 1/4× MIC tannic acid (TA) and vancomycin (VAN) on toxin production by C. difficile ATCC BAA-1870 (a, b) and BAA-1382 (c, d). Each experiment was repeated twice independently and included six biological replicates. Error bars represent standard error of the mean (SEM). The *P* value was obtained by one-way ANOVA.

As shown in Fig. 3, compared with the untreated control group and vancomycin-treated group, 1/2× MIC and 1/4× MIC TA treatments inhibited toxin production by ATCC BAA-1870 and BAA-1382 at three different time points. With regard to ATCC BAA-1870, except for 1/4× MIC TA treatment, no significant difference in TcdA inhibition was observed on day 1 of the experiment (versus control, *P = *0.119). At other time points, 1/2× and 1/4× MIC TA treatments presented statistically significant differences in the inhibition of TcdA and TcdB production by ATCC BAA-1870 (versus control, *P < *0.05). With respect to ATCC BAA-1832, 1/2× and 1/4× MIC TA treatments produced inhibitory effects on TcdA and TcdB production, presenting statistically significant differences (versus control, *P < *0.05). The control drug vancomycin failed to inhibit TcdA and TcdB production by C. difficile test strains.

### TA inhibits C. difficile biofilm formation.

**FIG 4:**
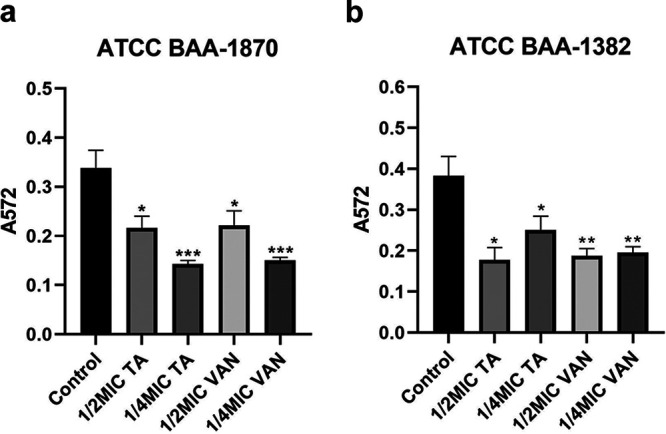
Effect of 1/2× MIC and 1/4× MIC tannic acid (TA) and vancomycin (VAN) on C. difficile BAA-1870 (a) and BAA-1382 (b) biofilm formation. Each experiment was repeated twice independently and included 16 biological replicates. Error bars represent standard error of the mean (SEM). *P* values were obtained by one-way ANOVA. *, *P < *0.05; **, *P < *0.005; ***, *P < *0.0001.

As illustrated in Fig. 4, the biofilm-forming ability of the two C. difficile test strains subjected to 1/2× and 1/4× MIC TA treatments was statistically different from that of the untreated control group (*P < *0.05), and the TA inhibitory effects were comparable to those of vancomycin. Moreover, the inhibitory effect of TA between the two C. difficile strains varied, with 1/4× MIC TA presenting a stronger inhibitory effect on ATCC BAA-1870 and 1/2× MIC TA exerting stronger inhibition on ATCC BAA-1382.

### TA inhibits the transcription of key genes for toxin production and sporulation in C. difficile.

**FIG 5:**
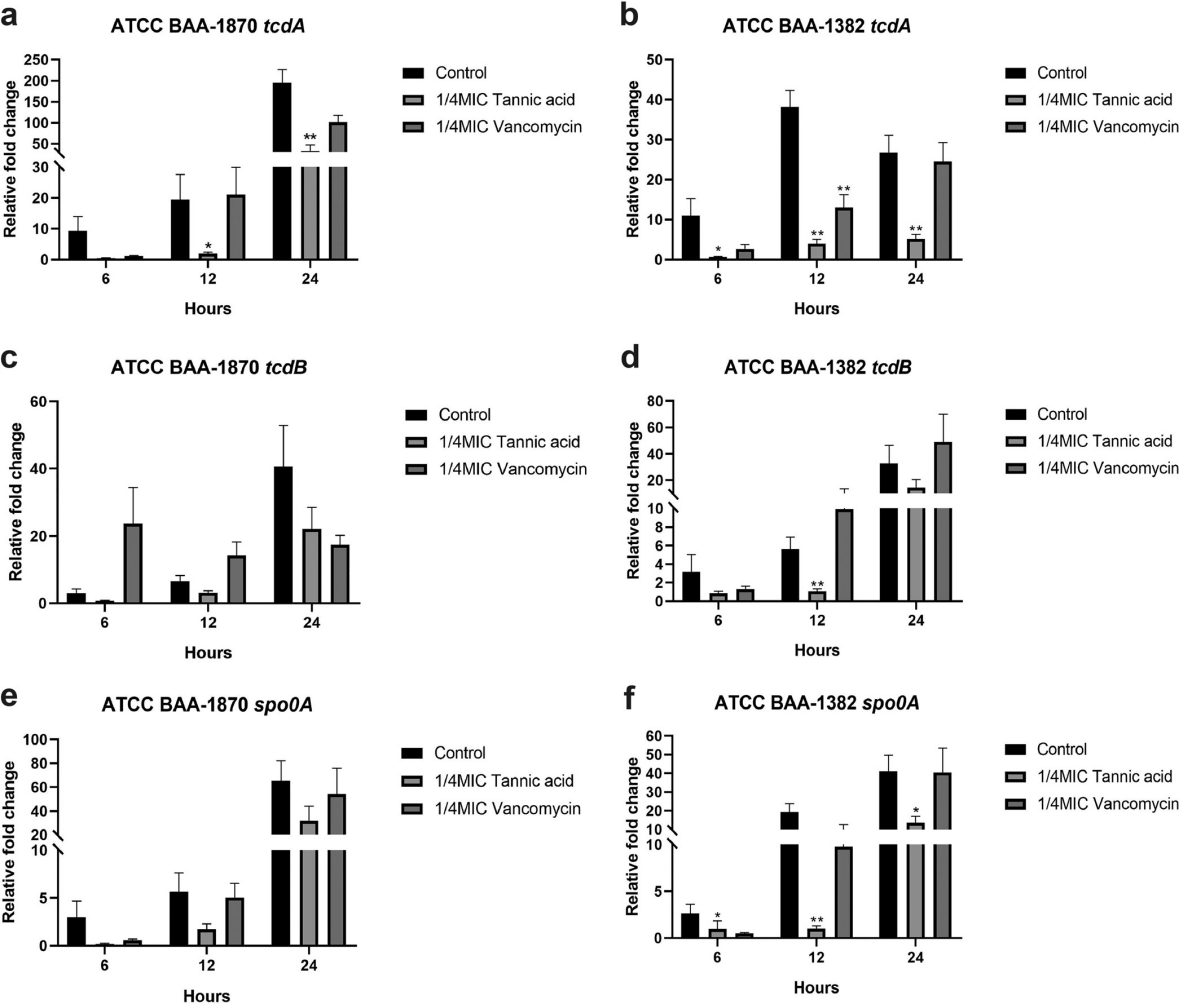
Effect of 1/4× MIC tannic acid (TA) and vancomycin (VAN) on the expression of *tcdA*, *tcdB*, and *spo0A* genes in C. difficile ATCC BAA-1870 (a, c, e) and BAA-1382 (b, d, f). Each experiment was repeated twice independently and included six biological replicates. Error bars represent standard error of the mean (SEM). *P* values were obtained by Mann-Whitney U test. *, *P < *0.05; **, *P < *0.005.

As indicated in Fig. 5, compared with the untreated control group, the mRNA expression of *tcdA*, *tcdB*, and *spo0A* of the 1/4× MIC TA and vancomycin treatment groups was inhibited, and the inhibitory effect of TA was stronger than that of vancomycin but differed between the strains. The expression of *tcdA* in ATCC BAA-1870 was inhibited at 6 h but was not statistically significant (*P = *0.180); however, *tcdA* expression was significantly inhibited by TA at 12 and 24 h (*P < *0.05). In ATCC BAA-1382, *tcdA* expression was significantly inhibited by TA at 6, 12, and 24 h (*P < *0.05). The expression of *tcdB* was inhibited in ATCC BAA-1870 at all the time points but did not exhibit any statistically significant difference (*P > *0.05). However, TA significantly inhibited *tcdB* in ATCC BAA-1382 only at 12 h (*P = *0.002) but inhibited *spo0A* in ATCC BAA-1870 at all time points without any statistically significant difference (*P > *0.05). In contrast, in ATCC BAA-1382, the inhibitory effect of TA on *spo0A* expression at each time point was statistically significant (*P < *0.05) (Fig. 5).

### TA reduces the weight of cecum in mice.

**FIG 6:**
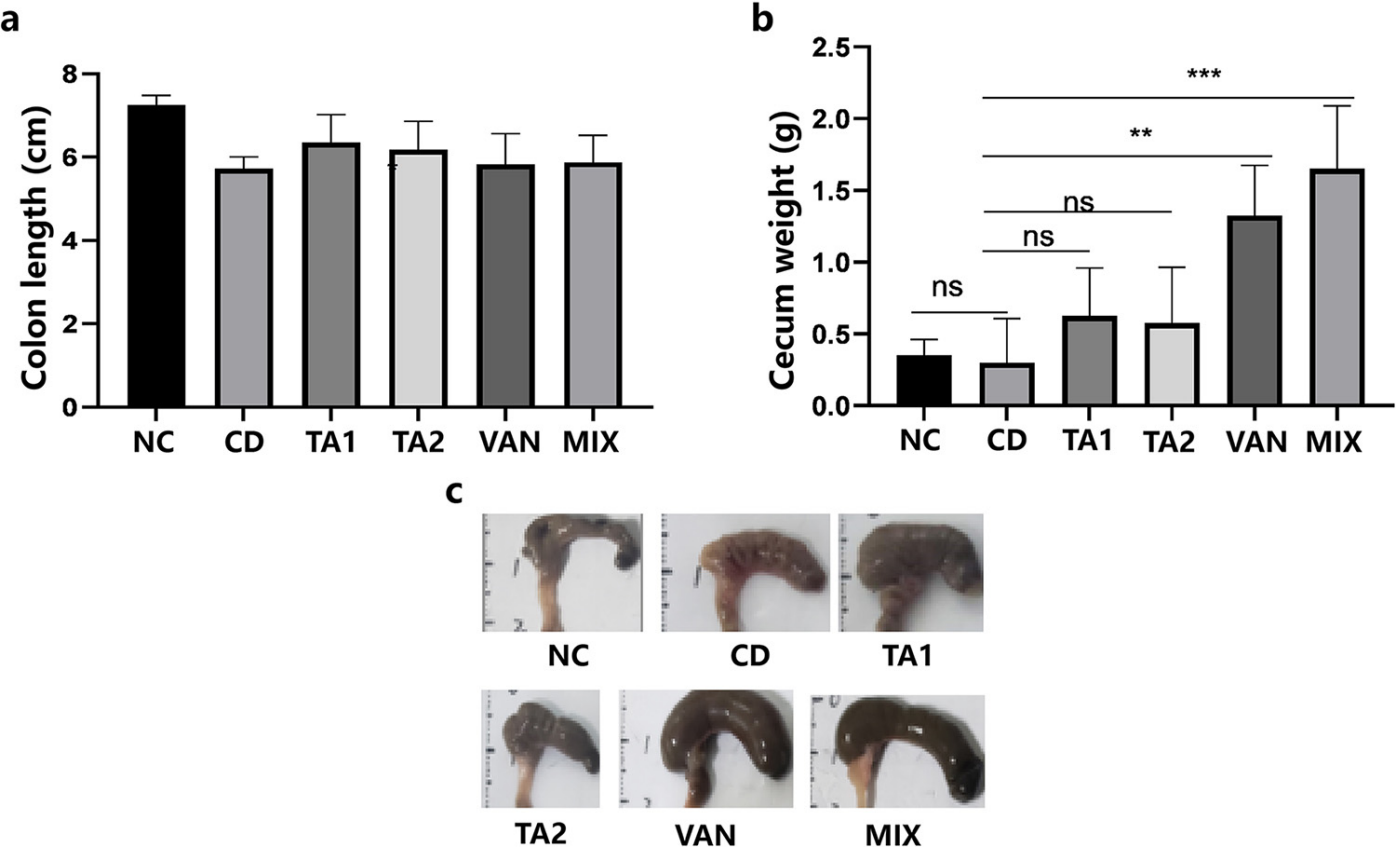
Colon length and cecum weight of each mice group measured after 5 days of treatment (*n* = 4). NC, nonchallenged control; CD, C. difficile infection control; TA1, 200 mg/kg tannic acid (TA) treatment; TA2, 100 mg/kg TA treatment; VAN, vancomycin treatment; MIX, vancomycin and TA treatment. (a) After 1 day of intragastric administration of the C. difficile suspension, 5 days after the start of treatment, the length of the colon of each group was measured. (b) After 5 days of treatment, the weight of the cecum in each group was measured. (c) After 5 days of treatment, the mice in each group were dissected, and the cecum was visualized. The error bars represent standard error of the mean (SEM).

As shown in Fig. 6a, after 5 days of treatment, no difference was noted in the length of the colon among the treatment groups (*P > *0.05). However, compared with the nonchallenged control (NC) group, the cecum in the VAN and mixed-treatment (MIX) groups was enlarged (*P < *0.01), whereas cecum swelling was not obvious after TA treatment (Fig. 6b). In the TA1 and TA2 groups, the weight of the cecum was not statistically significant compared with that in the NC group (*P > *0.05). Antibiotics can visually enlarge and increase the weight of the cecum, and the cecum of mice infected with C. difficile was hyperemic and swollen (Fig. 6c).

### Protective effect of TA on mice infected with C. difficile.

**FIG 7:**
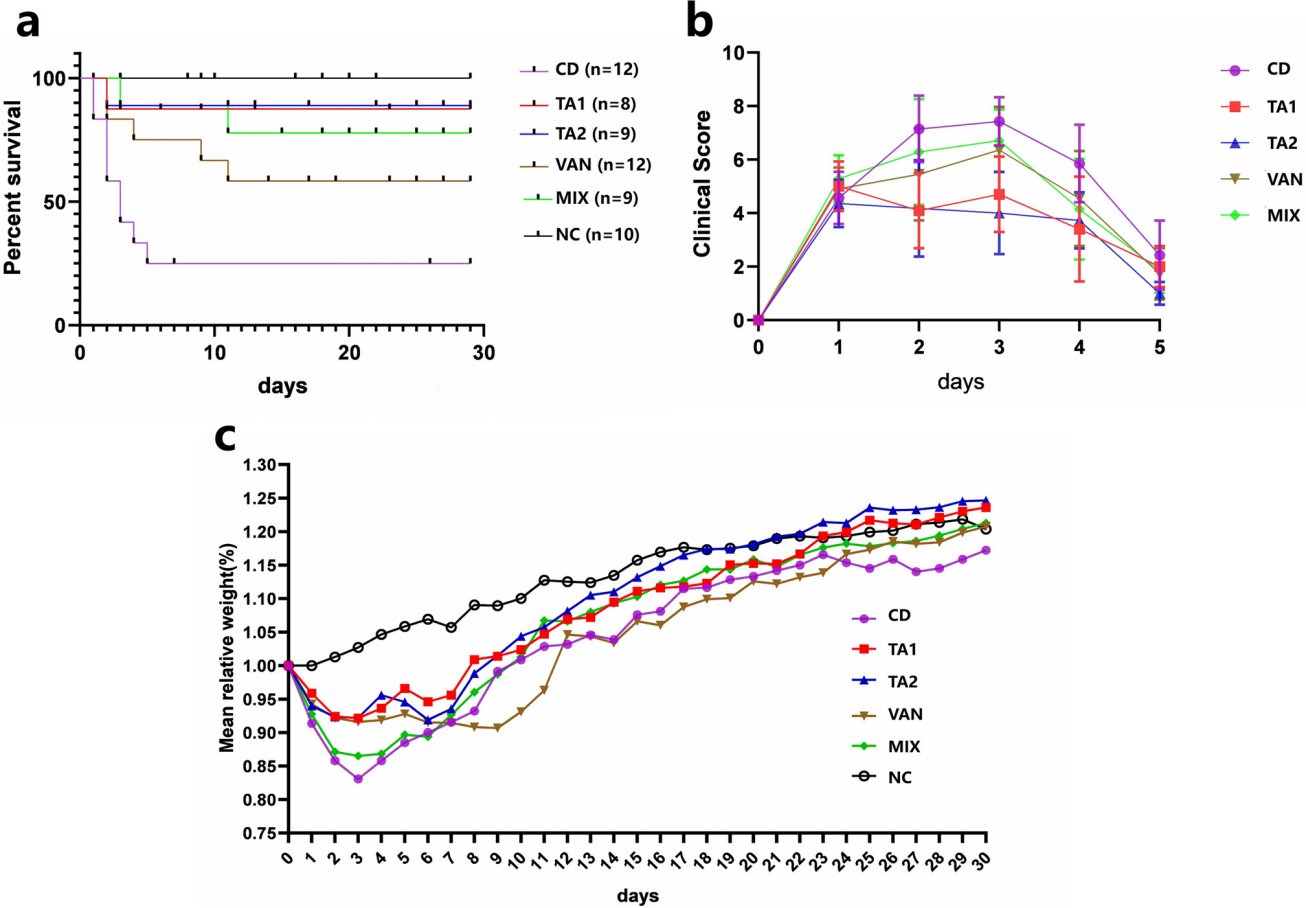
Protective effect of tannic acid (TA) on C. difficile-infected mice against CDI. NC, nonchallenged control; CD, C. difficile infection control; TA1, 200 mg/kg TA treatment; TA2, 100 mg/kg TA treatment; VAN, vancomycin treatment; MIX, vancomycin and TA treatment. TA treatment can protect mice from CDI. (a) Survival curve over 30 days. (b) Mouse clinical score over 5 days. (c) Relative percentage of body weight of surviving mice compared to day 0.

Mice that were exposed to antibiotics and then challenged with C. difficile developed clinical symptoms of CDI. In the CDI group (CD), the mice showed significant weight loss from day 2 and reached their lowest weight on day 3. By day 30, 75% of mice were dead (Fig. 7a and c). In the VAN group, the survival rate of mice was 75% on day 5, and their weights increased on days 4 and 5. After discontinuation of vancomycin, the animals developed signs of CDI and progressive weight loss from day 6, weight dropped to the lowest on day 9, and the survival rate was 58%. In the TA2 group, the body weight of the mice reduced in the first 3 days and then began to increase on day 4 and decreased to the lowest on day 7. The mice defecated soft stools when they lost weight, and no wet tail was noted. Subsequently, the body weight of mice slowly increased, and the survival rate was 89% on day 30. In the TA1 group, the body weight of the mice dropped to the lowest point on day 3, and the mice defecated wet stool. On day 6, the body weight of mice slowly increased, and the survival rate was 88% on day 30 (Fig. 7b and c).

### TA reduces the number of C. difficile spores.

**FIG 8:**
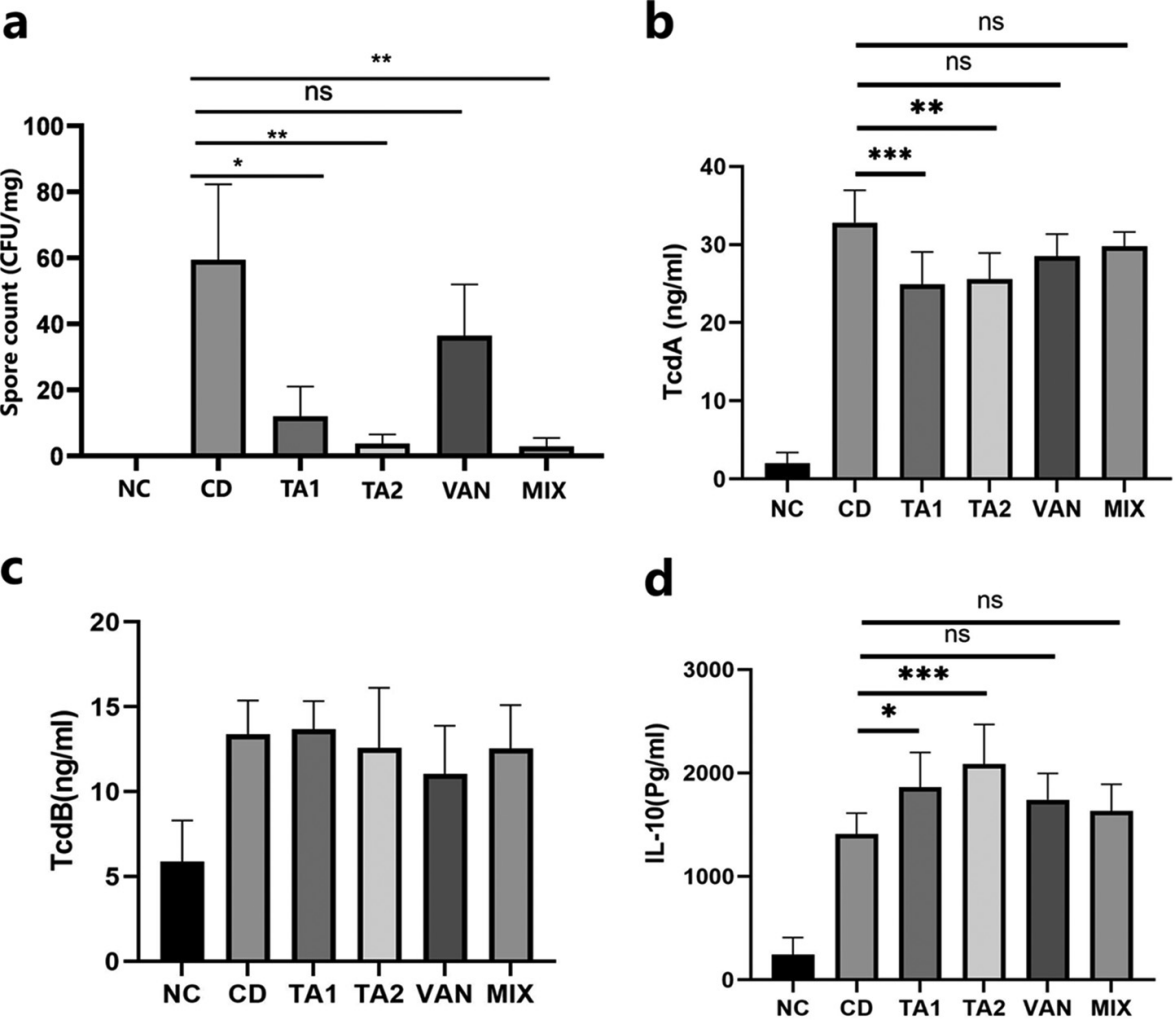
Inhibitory effect of tannic acid (TA) on C. difficile vegetative cell growth, spore formation, and TcdA and IL-10 expression in mice infected with C. difficile. NC, nonchallenged control; CD, C. difficile infection control; TA1, 200 mg/kg TA treatment; TA2, 100 mg/kg TA treatment; VAN, vancomycin treatment; MIX, vancomycin and TA treatment. (a) Spores in the cecal contents of mice (*n* = 4); (b) TcdA level in the serum of mice (*n* = 4); (c) TcdB level in the serum of mice (*n* = 4); (d) IL-10 level in the serum of mice (*n* = 4). The results are expressed as mean ± standard deviation. *, *P < *0.05; **, *P < *0.01; ***, *P < *0.001; ns, no statistical difference.

The number of spores in the feces of the two TA treatment groups was significantly reduced compared with that observed in the CD group. In contrast, no significant difference in the spore count was found between the VAN and CD groups (*P > *0.05) (Fig. 8a).

### TA reduces TcdA levels and increases IL-10 levels.

As shown in Fig. 8b, compared with the CD group, the TcdA content in the serum of TA1 and TA2 groups was significantly reduced. The serum TcdA levels in the VAN group (*n* = 4) and MIX group (*n* = 4) were not statistically significant compared with that in the CD group (*P > *0.05). In contrast, both treatment groups did not exhibit a decrease in serum TcdB levels compared with that in the CD group (Fig. 8c). Furthermore, the anti-inflammatory factor interleukin 10 (IL-10) in the serum of mice in the TA1 and TA2 groups was significantly increased (*P < *0.05) compared with that in the CD group, whereas the IL-10 levels in the serum of mice in the VAN and MIX treatment groups showed no statistical difference (*P > *0.05) (Fig. 8d).

### Histopathological changes in various treatment groups.

**FIG 9:**
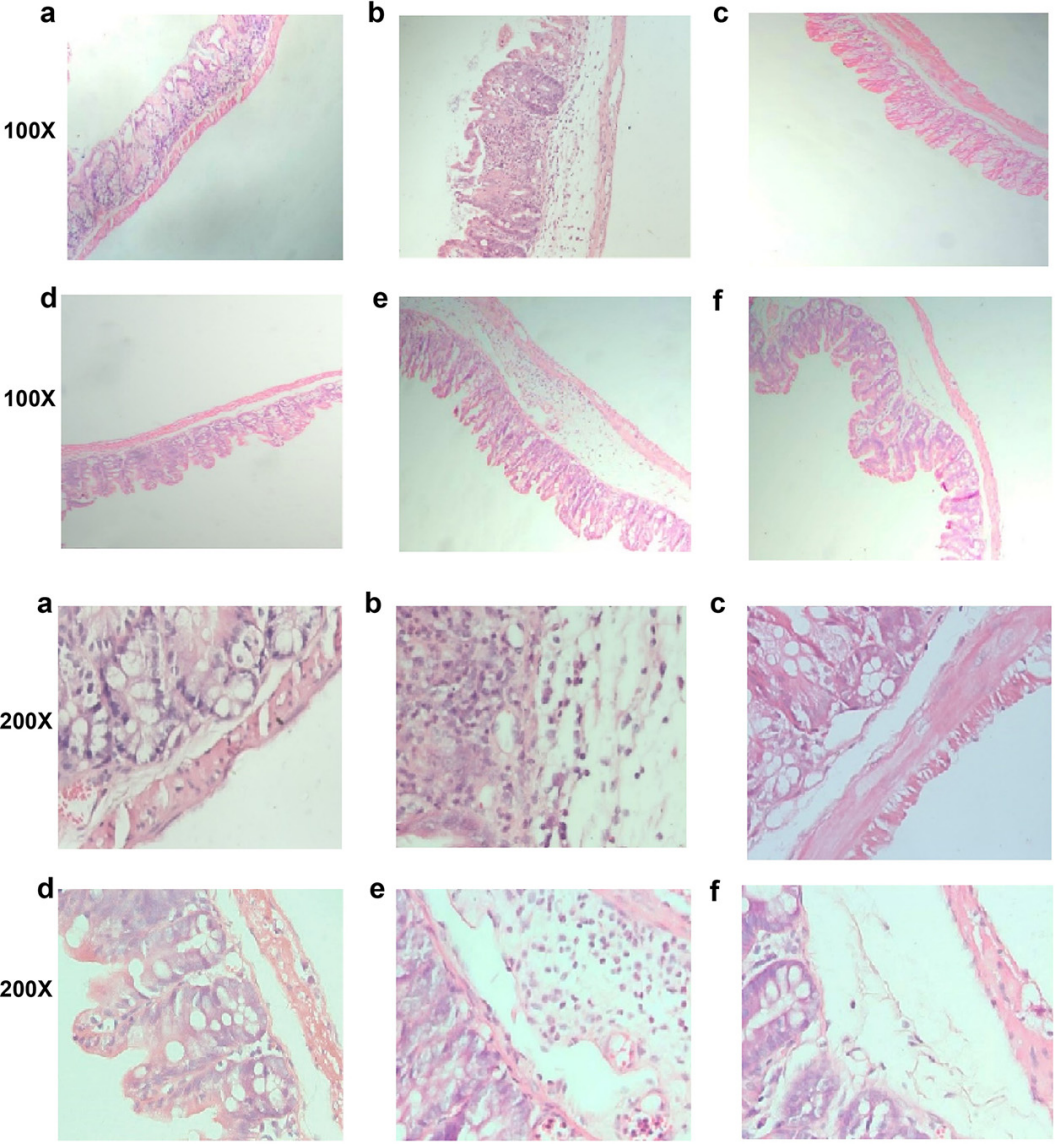
Histopathological analysis of the cecum of mice in each group after 5 days of treatment. (a) Nonchallenged control group (×100, ×200); (b) C. difficile infection control group (×100, ×200); (c) 200 mg/kg TA treatment group (×100, ×200); (d) 100 mg/kg tannic acid (TA) treatment group (×100, ×200); (e) Vancomycin treatment group (×100, ×200); (f) Vancomycin and TA treatment group (×100, ×200).

Analysis of the histopathological changes in the cecum tissue of mice revealed that TA can reduce intestinal inflammation (Fig. 9). Compared with the CD group, TA treatments significantly reduced submucosal edema and inflammatory cell infiltration (Fig. 9d and e). In the VAN and MIX groups, submucosal edema and neutrophils were still noted, compared with the NC group (Fig. 9c and f).

### Effects of TA on the composition of intestinal microbiota of mice.

**FIG 10:**
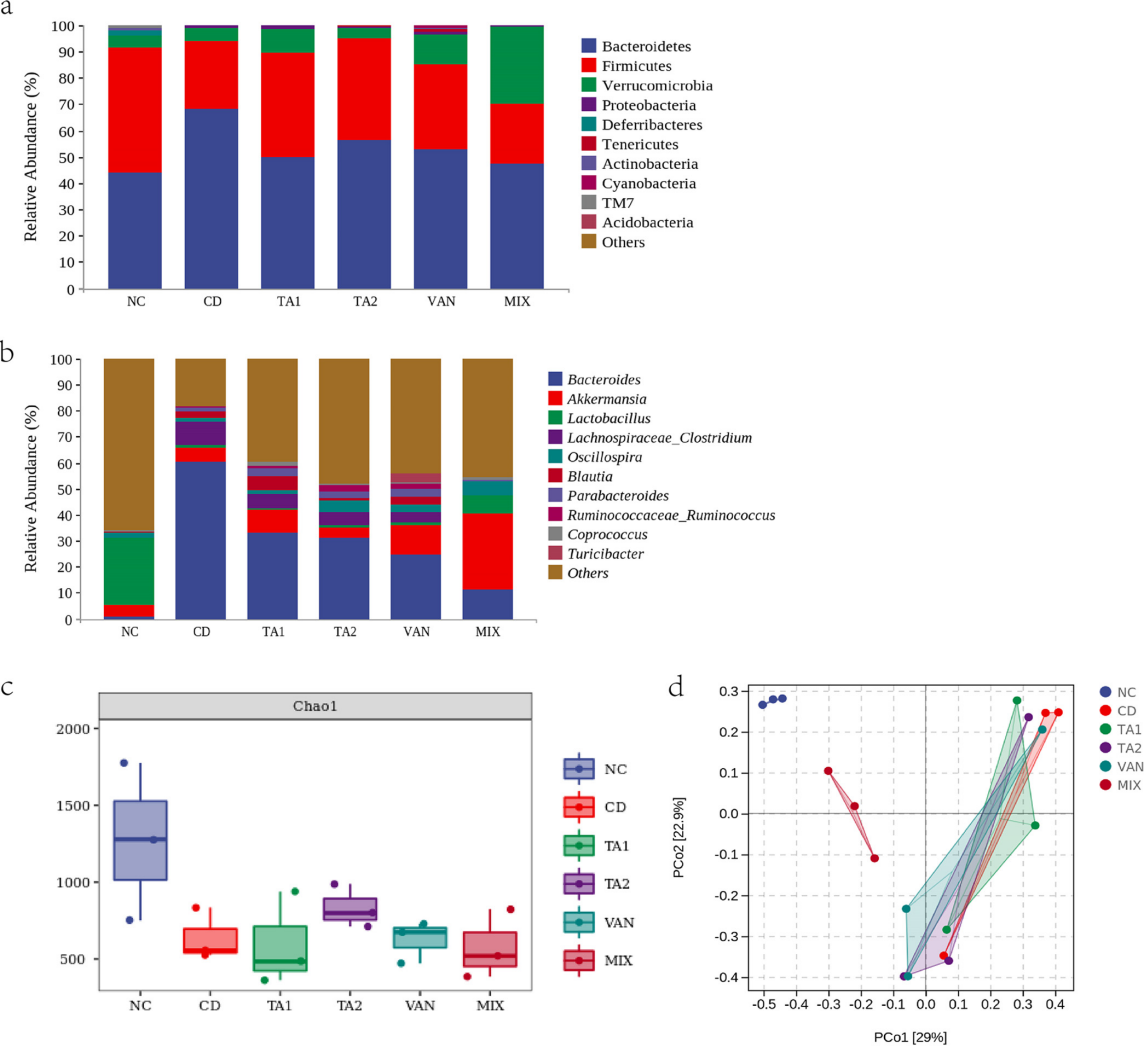
Effect of tannic acid (TA) on the composition of intestinal microbiota of mice. NC, nonchallenged control; CD, C. difficile infection control; TA1, 200 mg/kg TA treatment; TA2, 100 mg/kg TA treatment; VAN, vancomycin treatment; MIX, vancomycin and TA treatment. (a) Relative abundance of OTUs at the phylum level; (b) Relative abundance of OTUs at the genus level; (c) Chao1 index-evaluated α-diversity; (d) 00 principal-coordinate analysis (PCoA)–determined β-diversity.

At the phylum level, the composition of microbiota noted in the two TA groups was closer to that in the NC group compared with those in the CD and VAN groups. The mice in the VAN group showed reduced *Firmicutes* and increased *Verrucomicrobia*, whereas those in the CD group presented increased *Bacteroidetes* and reduced *Firmicutes*. The Chao1 index is an indicator of α-adversity. The α-diversity of the TA2 group was closer to that of the NC group compared with other treatments. The PCoA revealed that the β-diversity of each treatment group was different from that of the NC group (Fig. 10).

## DISCUSSION

In this study, we found the inhibitory effect of GC on C. difficile and explored the effect of its main component, TA, on CDI *in vitro* and *in vivo*. The results obtained showed that TA significantly inhibited C. difficile spore formation, toxin production, and biofilm formation *in vitro* and exerted a certain inhibitory effect on the *tcdA*, *tcdB*, and *spo0A* genes. Furthermore, TA was found to inhibit diarrhea, improve the survival rate of CDI mice, and alleviate disease severity in mice.

TA is a macromolecular phenolic compound that is extremely abundant in plants and is widely detected in gallnut, grape, tea, korshinski, pomegranate, and coffee. The polyphenolic hydroxyl structure of TA enables it to combine with polysaccharides, alkaloids, proteins, and metal ions to undergo complexation or electrostatic reaction (18). The molecular structure of TA is presented in Fig. S1 in the supplemental material. Many studies have shown that TA forms precipitate by complexing with the cell membrane proteins, phospholipids, and polysaccharides of pathogens, interferes with bacterial metabolism and nutrient absorption as well as self-oxidation to produce hydrogen peroxide, reduces the permeability of the cell membrane, and exhibits an inhibitory effect on pathogens (19). Clinical studies have proven the effectiveness of tannin gelatin in the treatment of diarrhea (20). Li et al. found that the TA-rich fraction from pomegranate rind (TFPR) exerted an antibacterial effect by destroying the integrity of the cell membrane of Listeria monocytogenes and causing imbalance in cell homeostasis (21). In addition, a medical product with TA as the main ingredient has been proven to exhibit a broad spectrum of effectiveness and safety in the treatment of diarrhea. The antidiarrheal effect of this product can be attributed to its ability to improve the intestinal epithelial barrier and capability to inhibit intestinal fluid secretion and high antioxidant capacity, thus strongly supporting the use of TA to prevent and treat CDI (22).

Spores are a kind of life form produced by certain bacteria in extreme environments which have strong resistance to heat, high pressure, drugs, radiation, oxidation, and other conditions (23). They play an important role in the transmission and pathogenicity of C. difficile, and their extremely high resistance makes them the major medium of disease transmission. Furthermore, owing to their resistance to antibiotics, therapeutic drugs cannot directly kill spores, causing the spores in the digestive tract to survive even after treatment (24). After treatment, the C. difficile spores are stimulated by the germinant in the intestine (cholate, amino acid, and Ca^2+^) and regerminate into vegetative cells, which can cause the infection to recur (22). Thus, inhibiting spore formation and allowing more bacteria to stay in the vegetative cell stage are crucial for the treatment of CDI (25). This study found that TA significantly inhibited C. difficile spore formation in a mouse model, which may be an important reason for the effectiveness of TA in CDI treatment in model mice (Fig. 8a and b).

The exotoxins TcdA and TcdB are the direct pathogenic factors of intestinal infection caused by C. difficile (26). Both TcdA and TcdB are encoded by the pathogenic region in the chromosome of C. difficile and expressed after being stimulated by the external environment (27, 28). In the present study, TA significantly inhibited the production of TcdA and TcdB, whereas the control drug, vancomycin, failed to show a similar effect *in vitro* (Fig. 4). Moreover, TA had an inhibitory effect on the serum TcdA content but not on the serum TcdB content, and the expression of IL-10 increased in the serum of mice treated with TA (Fig. 8). Also, TA has also been reported to alleviate the damage of the epithelial barrier caused by tumor necrosis factor (22). Thus, TA may have an inhibitory effect on the inflammatory response caused by CDI, which may be crucial to its therapeutic effect. It has been reported that antibiotics that exert a damaging effect on the bacterial cell wall accelerated the release of bacterial toxins, which aggravated the host inflammatory response (29). Also, the findings of the present study demonstrated that TA significantly inhibited biofilm formation, similar to vancomycin (Fig. 4). Biofilm is an important structure for microorganisms to adhere to and colonize the host surface. When the bacterial cells in the biofilm form spores, the biofilm becomes a storage space for spores, providing favorable conditions for the transformation of C. difficile acute infection to chronic and recurrent infection after treatment (30). Zhang et al. developed a biocompatible composite material with antibacterial and antibiofilm effects by combining TA and MgCl_2_ with bacterial cellulose. The composite material was found to have strong antibacterial activity and a certain antibiofilm effect against Staphylococcus aureus, Escherichia coli, and Pseudomonas aeruginosa (31).

It has been reported that the weight of the cecum increased and the cecum enlarged in shape after antibiotic treatment for CDI, which may be related to the disorder of the intestinal microbiota (32). Similarly, the results of the CDI model obtained in the present study are consistent with those reported in the literature after vancomycin treatment of C57BL/6N mice. As shown in Fig. 6c, although TA treatment aggravated cecal enlargement, no statistical difference was noted between the NC and TA treatment groups. Compared with the CD group, the TA treatment group exhibited beneficial effects in terms of survival rate, clinical score, and body weight, which were significantly better than those noted in the VAN and MIX groups (Fig. 7). In addition, histological experiment results showed that TA treatment significantly alleviated the inflammation of intestine, and its effect was better than that noted in other treatment groups (Fig. 9).

TA treatment produced a series of positive effects on CDI models, but the underlying mechanisms are unclear and must be further explored. These positive effects are presumed to be related to multiple mechanisms, including restoration of intestinal barrier function and increased concentrations of intestinal short-chain fatty acids (SCFAs), both of which are disrupted during CDI. It has been shown that TA treatment can increase the expression of intestinal tight junction protein genes ZO-1, ZO-2, and CLDN-2 and the SCFA concentration in the intestine (33, 34). In addition, studies have shown that SCFAs can not only directly inhibit C. difficile *in vitro* but also activate the host’s immune function and reduce the inflammatory response (35, 36). In the future, we will focus on restoring the intestinal barrier function and increasing the SCFA concentration to explore the specific mechanism.

However, the present study had the following limitations. In the *in vitro* experiments, only standard C. difficile strains were selected, and further verifications should be conducted on clinical strains. Moreover, although the effective therapeutic activity of TA was demonstrated in CDI mice models in the present study, owing to the huge physiological differences between mice and humans, clinical trials are still needed to prove the therapeutic effect of TA on human CDI. In addition, the preliminary study on intestinal microbiota was limited by sample size, and no significant effect of TA on the constitution of microbiota was observed; hence, future studies should include more samples. In conclusion, TA significantly inhibited C. difficile *in vitro* and had a potential therapeutic effect on CDI in mice models and thus could be applied as an alternative or auxiliary therapy for CDI treatment.

## MATERIALS AND METHODS

### Determination of MICs.

GC was purchased from Shijiazhuang Hospital of Traditional Chinese Medicine. A total of 130 strains of C. difficile were selected from the Hebei Provincial Bank for Medical Culture Collections (HBMCC) by random number table method. The GC was soaked in double-distilled water (ddH_2_O) for 40 min, decocted for 30 min, and filtered through a gauze, and the filtrate was collected. The remaining residue was decocted for another 20 min, the filtrate was collected, and, finally, the two filtrates were combined. Enhanced Brucella agar plates containing gradient concentrations of GC decoction were used to determine the MIC against C. difficile. The C. difficile suspension was inoculated onto the plate, and the results were read after 48 h of incubation at 37°C in an anaerobic environment. Subsequently, 20 mL of the decoction was dried in a sterile oven at 50°C and weighed, and the GC content in the agar containing each dilution ratio was calculated, which is the MIC of GC against C. difficile.

TA and GA from Chinese natural gallnuts were purchased from Sigma-Aldrich (St. Louis, MO, USA). The C. difficile suspension was inoculated onto enhanced Brucella agar containing gradient concentrations of the drugs and incubated at 37°C for 48 h in an anaerobic environment. After 48 h, the results were interpreted, and the lowest drug concentration without colony growth was defined as the MIC of the drug against the strains.

The MICs of TA and vancomycin against C. difficile ATCC BAA-1870 and ATCC BAA-1382 (both purchased from the American Type Culture Collection) were determined by the above-mentioned methods. Vancomycin was bought from National Institutes for Food and Drug Control (Beijing, China).

### Sporulation assays.

C. difficile, with an optical density at 620 nm (OD_620_) of 0.1, was cultured in brain heart infusion supplement (BHIS; supplemented with 0.5% yeast extract and 0.1% l-cysteine) to the early stationary phase (OD_620_, 0.45 to 0.5). Then, 100 μL of the culture (at an OD_620_ of 0.45 to 0.5) was added to 10 mL of BHIS (diluted at 1:100) and cultivated again to the early stationary phase. Subsequently, 1/2× or 1/4× MIC of TA or vancomycin was added to the culture, and 1 mL of the culture was collected every day for the next 5 days to quantify the heat-resistant spores. The collected 1-mL culture samples were heat shocked at 65°C for 30 min, 10-fold serially diluted, and inoculated onto enhanced Brucella agar plates containing sodium taurocholate to determine the spore numbers. The plates were incubated anaerobically for 48 h at 37°C. The obtained data were subjected to one-way analysis of variance (ANOVA) using SPSS version 21.0.

### Analysis of *in vitro* toxin production.

The test strains were inoculated into BHIS medium with or without drugs (1/2× MIC TA, 1/4× MIC TA, 1/2× MIC vancomycin, and 1/4× MIC vancomycin) and cultured at 37°C in an anaerobic environment. C. difficile TCD-A and TCD-B enzyme-linked immunosorbent assay (ELISA) quantitative kits (Jingmei, Jiangsu, China) were used to determine the amount of TcdA and TcdB in the culture supernatants at 1, 2, and 3 days of inoculation, respectively. The standard curve construction and ELISA were performed according to the manufacturer's instructions. The OD at 450 nm (OD_450_) was measured and compared with the linear range of the standard curve to calculate the total toxin concentration. The data obtained were subjected to one-way ANOVA using SPSS version 21.0.

### Quantification of biofilm formation.

The bacterial culture with OD_620_ of 0.5 was diluted to 1:100 and inoculated into 96-well plates containing 20 to 180 μL of BHISG medium (BHIS supplemented with 1% glucose, 0.5% yeast extract, and 0.1% l-cysteine) with or without drugs (1/2× MIC TA, 1/4× MIC TA, 1/2× MIC vancomycin, and 1/4× MIC vancomycin) and incubated anaerobically at 37°C for 48 h. After incubation, the wells were washed twice with sterile ddH_2_O and dried overnight. Then, the wells were stained with 200 μL of 0.2% crystal violet for 30 min and washed twice with sterile ddH_2_O. Subsequently, 200 μL of methanol was added to each well to decolonize for 30 min, and the OD_570_ was measured. The data were subjected to one-way ANOVA using SPSS version 21.0.

### Gene expression assay.

The test strains were inoculated into BHIS medium with or without drugs (1/2× MIC TA, 1/4× MIC TA, 1/2× MIC vancomycin, and 1/4× MIC vancomycin) and cultured anaerobically at 37°C. Then, 1 mL of the culture was collected to extract RNA at 0, 6, 12, and 24 h of incubation, respectively. The total RNA was isolated from the collected bacterial pellets using the bacterial RNA extraction kit (Omega Bio-tek; Norcross, GA, USA) according to the manufacturer’s recommendations. Subsequently, FastQuant reverse transcriptase (RT) kit (with gDNase) (Tiangen, Beijing, China) was used to remove genomic DNA and obtain cDNA by reverse transcription. Reverse transcriptase quantitative PCR (RT-qPCR) of *tcdA*, *tcdB*, and *spo0A* was performed using the published primers (see Table S1 in the supplemental material) and normalized against 16S rRNA gene expression. The reactions (25 μL) were performed in triplicate using SuperReal Premix Plus (SYBR green) (Tiangen, Beijing, China). The reaction cycles were as follows: 95°C for 15 min and 40 cycles of 95°C for 10 s, 55°C for 20 s, and 72°C for 30 s. The relative fold change in gene expression was calculated using the threshold cycle (2^−ΔΔ^*^CT^*) method (37). The sample mean *C_T_* of the 16S rRNA gene (internal control gene) was subtracted from that of *tcdA*, *tcdB*, and *spo0A* genes (Δ*C_T_*), and the Δ*C_T_* of the control taken at 0 h was subtracted from the mean Δ*C_T_* of each experimental sample (ΔΔ*C_T_*). This 2^−ΔΔ^*^CT^* method yielded the fold change in the expression of the gene of interest normalized to the expression of the 16S rRNA internal control gene and relative to the control taken at 0 h. Mann-Whitney U test was performed for statistical analysis of the data using SPSS version 21.0.

### Experimental animal.

A total of 60 specific-pathogen-free (SPF) male C57BL/6N mice (age, 4 to 5 weeks) were purchased from Beijing Weitong Lihua, China. The mice were raised in the SPF animal center of Hebei Medical University in dry and ventilated cages at 23°C to 25°C and 45% to 55% humidity. The animal experiment operation was strictly in accordance with the animal operation plan described in the animal ethics review. The study was approved by the Institutional Review Board of the Second Hospital of Hebei Medical University.

### CDI mouse model.

**FIG 11:**
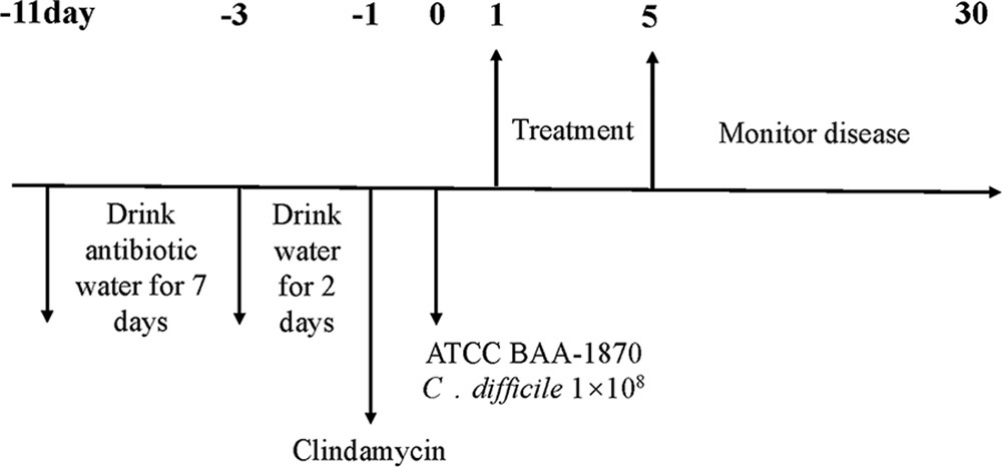
Mouse experiment flowchart.

Before the experiment, the mice were allowed to adapt to the environment for a week. One week later, an antibiotic mixture of polymyxin (0.1135 mg/mL), gentamicin (0.07 mg/mL), kanamycin (0.8 mg/mL), vancomycin (0.09 mg/mL), and metronidazole (0.43 mg/mL) was added to drinking water. Ten mice in each group were given water containing the antibiotic mixture for 7 days. Then, all mice were given regular autoclaved water for 2 days and a single dose of clindamycin (100 mg/kg of body weight) intraperitoneally for 1 day before C. difficile challenge. Subsequently, the suspension of C. difficile ATCC BAA-1870 (1 × 10^8^ CFU/mouse) was administered by gavage. After oral administration of the C. difficile suspension for 24 h, the mice were treated for 5 days and monitored continuously for 30 days. The body weight was recorded every day, and clinical scores were given for 5 consecutive days. According to the requirements of the IDSA clinical score sheet, mice with a weight loss of 25% after infection were euthanized (Fig. 11).

### Experimental scheme.

To establish a mouse model of CDI and evaluate treatment, 60 mice were randomly divided into 6 groups according to random block method of body weight. As shown in Fig. 11, in the nonchallenged control (NC) group (*n* = 10), the mice received regular food and water without any treatment. The remaining five groups were model groups, the CDI group (CD) (*n* = 12), consisting of control-infected mice receiving pretreatment with a 7-day course of antibiotic mixture and C. difficile challenge; the vancomycin treatment group (VAN) (*n* = 12), consisting of infected mice treated with vancomycin (50 mg/kg/day) for 5 days; the TA treatment group 1 (TA1) (*n* = 9), consisting of infected mice treated with 200 mg/kg/day TA for 5 days; the TA treatment group 2 (TA2) (*n* = 8), consisting of infected mice treated with 100 mg/kg/day TA for 5 days; and the mixed-treatment group (MIX) (*n* = 9), consisting of infected mice treated with vancomycin (50 mg/kg/day) and TA (200 mg/kg/day) for 5 days. All the groups were observed until the end of the experiments.

### C. difficile spore load in cecal contents.

At the end of the experiment (day 30), the mice were dissected, and their intestinal contents (20 mg/mouse) were collected into Eppendorf tubes, treated with absolute ethanol for 1 h, and centrifuged. The precipitate obtained was dissolved in 400 μL of BHI, followed by continuous dilution 10 times. To determine the spore number, the samples were first heat shocked (65°C for 30 min) to kill any vegetative cells and then inoculated onto a Clostridium difficile moxalactam norfloxacin (CDMN) agar (Oxoid, Basingstoke, UK) with a 0.1% sodium taurocholate plate and incubated anaerobically for 48 h at 37°C. Each specimen was inoculated in triplicate, and the average of the plate count was determined. One-way ANOVA of the obtained data was performed using SPSS version 21.0.

### Quantification of TcdA, TcdB, and IL-10 production in mice.

After the mice were treated for 5 days, mice were euthanatized to collect the blood, which was centrifuged at 2,500 rpm for 10 min to obtain serum. Subsequently, the absorbance was measured at 450 nm, and the obtained data were subjected to one-way ANOVA using SPSS version 21.0.

### Cecum histopathological score.

To assess the histopathological score, 60 mice were divided into six groups (NC, CD, TA1, TA2, VAN, and MIX groups). After day 5 of treatment, the mice were sacrificed by cervical dislocation. The cecum was dissected and fixed with 4% paraformaldehyde. After 24 h, the cecum was transferred to 75% alcohol for long-term storage. Then, 6-μm-thick cecum sections were stained with hematoxylin and eosin (H&E) for histological analysis, and the tissues were reviewed in a blinded fashion. The obtained data were subjected to one-way ANOVA by using SPSS version 21.0.

### Fecal DNA extraction, PCR amplification, and sequencing.

Fresh feces from mice were collected by stress method on day 30, and the changes in the abundance and diversity of the intestinal microbiota of mice after each treatment were analyzed. The total genomic DNA samples were extracted using Omega soil DNA kit (catalog no. M5635-02; Omega Bio-tek). A NanoDrop NC2000 spectrophotometer (Thermo Fisher Scientific, Waltham, MA, USA) was employed to quantify the DNA, and the quality of the extracted DNA was verified using 1.2% agarose gel electrophoresis. PCR amplification of the V3-V4 region of the bacterial 16S rRNA gene was performed using the primers 338F (5′-ACTCCTACGGGAGGCAGCA-3′) and 806R (5′-GGACTACHVGGGTWTCTAAT-3′). Sample-specific 7-bp barcodes were incorporated into the primers for multiplex sequencing. *Pfu* high-fidelity DNA polymerase (Quanshijin Co., China) was used for PCR amplification, and the amplified results were subjected to 2% agarose gel electrophoresis. The target fragments were cut out and purified with Vazyme VAHTSTM DNA clean beads (Vazyme, Nanjing, China). The PCR products were quantified using Quant-iT PicoGreen double-stranded DNA (dsDNA) assay kit (Invitrogen, Carlsbad, CA, USA).

After the individual quantification step, the amplicons were pooled in equal amounts, and the sequencing library was prepared using TruSeq Nano DNA low-throughput (LT) library prep kit from Illumina (San Diego, CA, USA). Before sequencing on the computer, quality inspection of the library was performed using an Agilent bioanalyzer (Agilent, Santa Clara, CA, USA). After passing quality inspection, Quant-iT PicoGreen dsDNA assay kit was employed to quantify the library on a Promega QuantiFluor fluorescence quantification system (Promega, Madison, WI, USA), and paired-end 2 × 250-bp sequencing was performed using Illumina NovaSeq platform with NovaSeq 6000 SP reagent kit (500 cycles) at Shanghai Personal Biotechnology Co., Ltd (Shanghai, China).

### Bioinformatics analysis.

The original data were trimmed and quality filtered, and the original sequence that passed the preliminary quality screening was divided into the library and sample according to the index and barcode information, and the barcode sequence was removed. The raw sequence data were demultiplexed using the demux plugin, followed by primer cutting with the cutadapt plugin (38). Then, the sequences were quality filtered, denoised, and merged, and chimeras were removed using DADA2 plugin (39). Nonsingleton amplicon sequence variants (ASVs) were aligned with mafft and used to construct a phylogeny with fasttree2 (40, 41). Taxonomy was assigned to ASVs using the classify-sklearn naive Bayes taxonomy classifier in feature-classifier plugin against the Greengenes Database (42, 43). The relative abundance of ASVs and Venn diagram were analyzed to obtain the difference or shared information of bacterial species abundance and ASVs between the groups. At the same time, the specific composition of each group at the taxonomic level of different species was determined. According to the distribution of ASVs in each group, the α-diversity level was evaluated. In addition, the distance matrix of each sample was calculated, and principal-coordinate analysis (PCoA) was performed to measure the difference in β-diversity between the groups.

### Data availability.

The metagenomic sequences of intestinal microbiota are available in the NCBI Sequence Read Archive (SRA) (http://www.ncbi.nlm.nih.gov/sra) under BioProject accession number PRJNA867564.
