# Supplementary material for: Antimicrobial Activity of Tannic Acid In Vitro and Its Protective Effect on Mice against Clostridioides difficile
Source: Microbiol Spectr. 2022 Dec 20;11(1):e02618-22. doi: 10.1128/spectrum.02618-22 (PMC9927261; doi:10.1128/spectrum.02618-22)
Supplement: Supplemental file 1 — Supplemental material. Download spectrum.02618-22-s0001.pdf, PDF file, 0.2 MB [file spectrum.02618-22-s0001.pdf]

Supplementary Table S1 Primers used in real-time PCR

Gene	Primer	Sequence （5′ –3′）
tcdA	tcdA-F	CAACACCTTAACCCAGCCATA
	tcdA-R	AGAGTTTTCTGCGGTAGCTGA
tcdB	tcdB-F	ATCTGGAGAATGGAAGGTGGT
	tcdB-R	TGATGGTGCTGAAAAGAAGTG
spo0A	spo0A-F	AGCGCAATAAATCTAGGAGCA
	spo0A-R	AGGTTTTGGCTCAACTTGTGT
16S rRNA	16S-F	AGCGGTGAAATGCGTAGATAT
	16S-R	CAGCGTCAGTTACAGTCCAGA

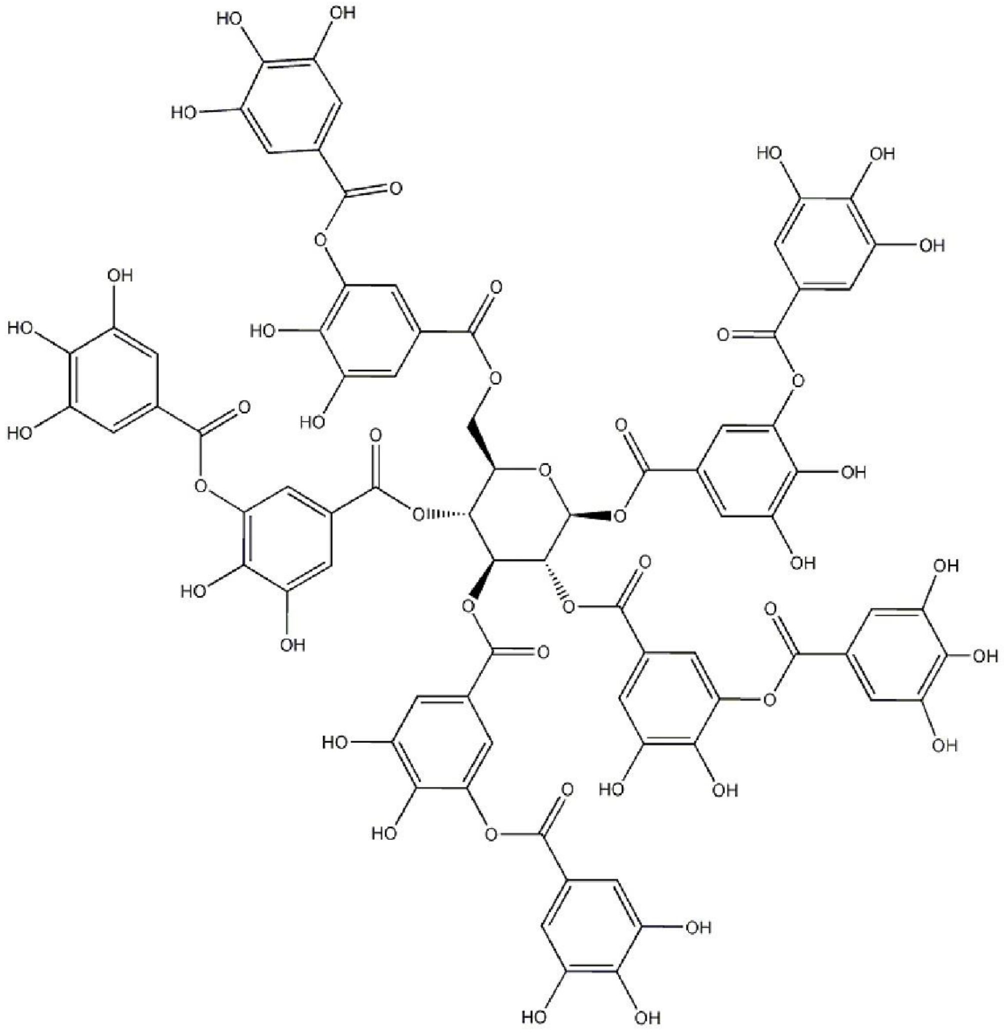

Supplementary Figure S1. Molecular structure of tannic acid.
